# Effect of low salicylate diet and blood salicylate level on the symptom control of chronic spontaneous urticaria

**DOI:** 10.3389/falgy.2025.1687600

**Published:** 2025-12-08

**Authors:** Sercan Guloglu, Ayse Bilge Ozturk, Said Incir, Betul Buyuktiryaki, Asli Gelincik, Semra Demir, Ebru Arik Yilmaz, Pinar Uysal, Mustafa Arga, Ozlem Cavkaytar, Reyhan Gumusburun, Tugba Gokce, Merve Poyraz, Ayse Baccioglu, Emek Kocaturk, Tuba Reçber, Emirhan Nemutlu, Cansin Sackesen

**Affiliations:** 1 Koc University Research Center for Translational Medicine (KUTTAM), Istanbul, Türkiye; 2 Immunology, Graduate School of Health Sciences, Koc University, Istanbul, Türkiye; 3 Division of Allergy and Immunology, Department of Pulmonary Medicine, School of Medicine, Koc University, Istanbul, Türkiye; 4 Department of Clinical Biochemistry, School of Medicine, Koc University, Istanbul, Türkiye; 5 Division of Pediatric Allergy, School of Medicine, Koc University, Istanbul, Türkiye; 6 Division of Immunology and Allergic Diseases, Department of Internal Medicine, Faculty of Medicine, Istanbul University, Istanbul, Türkiye; 7 Division of Pediatric Allergy, Faculty of Medicine, Pamukkale University, Denizli, Türkiye; 8 Division of Pediatric Allergy and Immunology, School of Medicine, Adnan Menderes University, Aydin, Türkiye; 9 Division of Pediatric Allergy and Immunology, Faculty of Medicine, Istanbul Medeniyet University, Istanbul, Türkiye; 10 Division of Allergy and Immunology, Department of Internal Medicine, Faculty of Medicine, Ege University, Izmir, Türkiye; 11 Division of Pediatric Endocrinology and Diabetes, Koc University Hospital, Istanbul, Türkiye; 12 Division of Allergy and Immunology, Department of Internal Medicine, Faculty of Medicine, Kirikkale University, Kirikkale, Türkiye; 13 Department of Dermatology, School of Medicine, Koc University, Istanbul, Türkiye; 14 Department of Analytical Chemistry, Faculty of Pharmacy, Hacettepe University, Ankara, Türkiye

**Keywords:** chronic spontaneous urticaria, low salicylate diet, salicylic acid, salicylate, urticaria

## Abstract

**Background:**

Up to 30% of chronic spontaneous urticaria (CSU) patients and 24% of children with CSU may have an NSAIDs-exacerbated cutaneous disease (NECD). Some vegetables and fruits are rich in salicylate. Salicylates in food can exacerbate symptoms in CSU patients.

**Aim:**

Our aim is to investigate the effect of a low salicylate diet on urticaria severity, quality of life, blood salicylate level and urine arachidonic acid pathway metabolites.

**Methods:**

Patients followed a fourweek low salicylate diet. Chronic urticaria quality of life questionnaire (CU-Q2oL) and 4 Days-Urticaria Activity Scores (UAS4) were recorded and blood and urine samples were collected at baseline and after the low salicylate diet. Urine Leukotriene-E4, Prostaglandin-E2, Prostaglandin-F2*α*, Thromboxane-A2, and creatinine levels were measured via ELISA. Blood salicylate level was determined by LC-MS/MS.

**Results:**

A total of 36 CSU patients were included in the study. The CU-Q2oL scores significantly decreased from 33.7 to 20.7 (*p* < 0.001) and the UAS4 significantly decreased from 14 to 8 (*p* < 0.001) after low salicylate diet when compared to baseline (low scores mean less complaints). The blood salicylate level was significantly lower after the low salicylate diet compared to the baseline (*p* = 0.042). However, there was no significant effect of the diet on urinary LTE4, PGDE2, PGDF2α and TXA2 levels.

**Conclusion:**

Our findings suggest that a low salicylate diet may help to reduce the severity of urticaria and improve quality of life by lowering blood salicylate levels. However, the diet had no impact on urinary LTE4, PGDE2, PGDF2α, and TXA2 levels.

## 1 Introduction

Chronic spontaneous urticaria (CSU) is an inflammatory skin disease manifested by the occurrence of spontaneous wheals or/and edema for more than six weeks (1). Its prevalence is estimated as 1% in the general population (2). Nonsteroidal anti-inflammatory drugs (NSAIDs) are the most frequently consumed drugs worldwide and NSAID hypersensitivity has been reported as the most common trigger of drug-induced cutaneous allergic reactions (3). It has been estimated that up to 30% of CSU patients and 24% of children with CSU may have an NSAIDs-exacerbated cutaneous disease (NECD) (3–5).

Salicylates are a group of chemicals derived from salicylic acid (6). They are found naturally in certain foods and manufactured for use in products such as aspirin, toothpaste, and food preservatives (6). Natural salicylates are found in a wide array of foods, including fruits, vegetables, nuts, and spices. Salicylate is especially high in alcoholic beverages, herbs, spices and non-alcoholic beverages including fruit juices, and tomato-based sauces (7). Spices including mint and red pepper contain the highest percentage of natural salicylates with 54.20 mg/kg and 28.25 mg/kg, respectively (7). The foods high in salicylates also include legumes (e.g., lentils, beans), vegetables (e.g., cauliflowers, pickled vegetables) and fruits (e.g., strawberries, plums, watermelons, raspberries) and some cereals (e.g., buckwheat, oat, corn ) (6). Daily salicylate intake can vary between 0.4–200 mg/day depending on the type of diet (7). Median concentrations of salicylate levels in serum samples from vegetarians and non-vegetarians were found as 0.11 µmol/L and 0.07 µmol/L, respectively while the median serum concentration of salicylic acid in patients taking 75 mg daily aspirin was 10.03 µmol/L (8). However, in cell culture studies, it has been shown that salicylic acid can inhibit cyclooxygenase pathway even at very low concentrations such as 0.1 µmol/L (9).

Foods that are high in salicylate may activate mast cells and trigger urticaria like aspirin hypersensitivity. However, the role of dietary salicylate is underestimated. In a subset of patients, pseudoallergens which include preservatives, dyes, aromatic compounds and salicylates found in both natural and processed foods may induce or worsen CSU (10). It has been shown that diets free from pseudoallergens may reduce urticaria symptoms (10, 11). However, the efficacy of dietary modifications in the treatment of CSU is controversial. A recent non-randomized clinical study showed that the low salicylate diet significantly decreased the itch scores in CSU patients after a 2–4-week diet (6). In certain populations, where the Mediterranean diet -rich in salicylate containing foods- is commonly followed, there may be a link between dietary habits and the worsening of CSU symptoms. Therefore, we aimed to study the effect of low salicylate diet on urticaria severity and quality of life by measuring blood salicylate level and urine arachidonic acid pathway metabolites in CSU patients including children and adults.

## 2 Method

### 2.1 Study design and study participants

**Figure 1:**
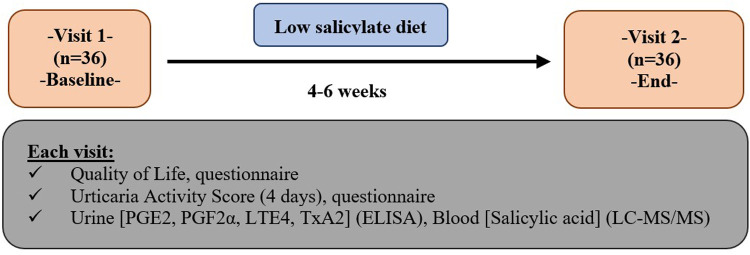
Study design.

A prospective, multicenter non-randomized, baseline-controlled intervention study was conducted. The study was approved by the ethics committee of Koc University and all subjects provided written informed consent. A detailed scheme of the study is shown in Figure 1. The participants meeting the following criteria were included in the study: CSU patients with uncontrolled urticaria under their current urticaria treatment such as second-generation antihistamines up to four-fold of antihistamine or omalizumab at a fixed dose of 300 mg/month from eight different Allergy and Immunology centers. *All patients had been on maximum-dose treatment for at least four weeks. Disease control was assessed by asking patients the following question: “Overall, how well have your treatments controlled your urticaria during the past four weeks?” Possible responses were: No response, Partial response, Well response to the treatment. Patients who responded Not at all, A Partial Response were considered to have uncontrolled disease and were included in the study. Those who reported being Well controlled were excluded.*

Exclusion criteria were as follows: Pregnancy, age below 6 years, concomitant chronic conditions such as heart failure, hypertension etc., use of immunosuppressant drug, inducible urticarias, acute urticaria, history of food allergies, use of aspirin or NSAID.

### 2.2 Dietary intervention

A low salicylate diet was an elimination diet that consisted of avoiding foods rich in salicylates. Foods containing high salicylate levels were determined from the literature and excluded from the diet for 4 weeks. The low salicylate diet has been prepared by a dietitian based on daily basic needs and age. Recipes for the low-salicylate diet were provided along with a daily meal plan. Patients were also informed about salicylate-containing cosmetic products, creams, gels, medicines and vegetable oils, etc., and it was recommended to check the content of each product before use and to avoid products containing salicylate.

A food diary was given to the patients, and each patient was asked to record the foods they frequently consumed, the cosmetic products and herbal oils they used that could potentially contribute to high salicylate intake. The patients continued their CSU treatment consisting of antihistamine or omalizumab, however no step-up regimen or rescue antihistamines were provided during the study period. At baseline (Visit-1) and after the low salicylate diet (Visit-2) all participants completed the CU-Q2oL and UAS4 questionnaire, blood and urine samples were collected at both time points to assess changes in blood salicylate levels and urinary levels of leukotrienes (LTE4), prostaglandins (PGDE2, PGDF2*α*) and thromboxane (TXA2).

### 2.3 Autologous serum skin test

About 5 mL of venous blood was collected in a sterile vacutainer and allowed to clot at room temperature for 30 min. Serum was centrifuged at 450–500 g for 10 min, and 0.05 mL of autologous serum was injected intradermally after cleaning the volar forearm skin with an antiseptic. Similarly, 0.05 mL of 0.9% sterile normal saline was injected intradermally as a negative control. Skin prick test with 10 mg/mL histamine was used as a positive control. A serum induced erythematous weal with a diameter of 1.5 mm more than the saline induced response within 30 min was considered positive (12).

### 2.4 The urticaria activity score

The urticaria activity score (UAS) given in Supplementary Table S1 was used to assess disease activity (1). Total score for the UAS was the sum of the score (0–3 for wheals and 0–3 for pruritus) for each day for 4 days with a maximum score of 24. For the convenience of study, it was preferred to use UAS4 (13).

### 2.5 Chronic urticaria quality of life (CU-Q2oL) score

The validated version of the Chronic Urticaria Quality of Life (CU-Q2oL) questionnaire was used to assess the quality-of-life impairment (14, 15). The CU-Q2oL is a 23-item questionnaire measures six dimensions (scales) of Health Related (HR) quality of life using a five-point (0–4) Likert-type scale: “pruritus” (2 items), “swelling” (2 items), “impact on life activities” (6 items), “sleep problems” (5 items), “limits” (3 items) and “looks” (5 items). Total score across all questions was calculated and transformed into percentile, ranging from 0 to 100, with a score of 100 indicating the worst possible quality of life impairment.

### 2.6 Measurements urine leukotriene and prostaglandin levels

Concentrations of leukotrienes (LT), prostaglandins (PG) and thromboxane (TX) including LTE4, PGE2, PGF2α, and TXA2 measured by commercially available enzyme-linked immunosorbent assays, with Human PGE2 ELISA kit, Human PGF2α ELISA kit, and Human LTE4 ELISA kit (from Cayman Chemical Company, Ann Arbor, MI, U.S.A.), Human TXA2 ELISA kit (Langham Creek Dr, Houston, U.S.A). The obtained levels of markers were normalized to urinary creatinine concentrations, measured with Cobas 6,000 autoanalyzer (Roche, Mannheim, Germany), and the results were expressed as pg/mL.

### 2.7 Measurement of plasma salicylic acid levels

Plasma salicylic acid (SA) was quantified by LC-MS/MS using SA-d₄ as the internal standard. Stock solutions (1 mg/mL in acetonitrile–water, 1:1) were stored below −18°C. Calibration standards (0.015–1.00 µg/mL) were prepared in blank plasma. For extraction, 100 µL plasma was mixed with 200 µL acetonitrile containing SA-d₄ (1 µg/mL), vortexed, and centrifuged (15,000 rpm, 10 min). The supernatant was analyzed on a Shimadzu LC-20AXR/8030 system with an Atlantis C18 column (4.6 × 75 mm, 3 µm) and C18 guard cartridge at 40°C. The mobile phase consisted of 0.1% formic acid (A) and acetonitrile (B) with a gradient of 40% B (0–0.5 min), 75% B (0.5–3.0 min), and re-equilibration to 40% B (3.0–6.0 min). Flow rate was 400 µL/min, injection volume 20 µL. Detection was in negative ESI mode using MRM transitions 137 → 93 m/z (SA) and 141 → 96.9 m/z (SA-d₄).

### 2.8 Statistical analysis

Data distribution was assessed using both Kolmogorov–Smirnov and Shapiro–Wilk tests. The Wilcoxon signed-rank non-parametric paired test was used for comparisons between two groups. P-value lower than 0.05 was accepted as significant.

## 3 Results

### 3.1 Study group and baseline findings

Thirty-six subjects [24 female (67%)] were recruited for the study. Demographic characteristics of the participants are given in Table 1. All the analyses were performed for the participants who completed both visit 1 and visit 2. The median ages for the study group were 15.3 (12.8–30.9) (median, interquartile range) years, 39.7 years (26.9–47.6) for adults (*n* = 12) and 13.3 (11.4–15.5) years for children (*n* = 24). Participants had been suffering from CSU for 7.9 (4.8–29.9) months until they visited the clinic. The weekly symptom attack frequency was 7 (median). Fifty-eight percent of patients reported to have angioedema while 64% reported stress as a triggering factor. The atopy rate was 18%. Twenty-nine percent of the participants were positive for dermographism. The autologous serum skin test positivity rate was 53%. Mean serum total IgE level of the participants (*n* = 31) was 62.8 (23.0–141.0) IU/mL.

**Table 1:** Demographic characteristic of the study participants (*n* = 36) who completed both baseline (visit 1) and after low salicylate diet (visit 2).

Characteristics		*n*, %
Sex (female)(%), (*n* = 36)		24 (67)
	(all participants)(years)*, (*n* = 36)	15.3 (12.8–30.9)
Age	(children: <18)(years)*, (*n* = 24)	13.3 (11.4–15.5)
	(adults: ≥18)(years)*, (*n* = 12)	39.7 (26.9–47.6)
Disease duration (months)*, (*n* = 35)		7.9 (4.8–30.0)
Attack frequency (weekly)*, (*n* = 35)		7.0 (4.0–7.0)
Atopic Sensitization, *n* (%), (*n* = 22)		4 (18)
Dermographism )(%), (*n* = 34)		10 (29)
Total IgE (IU/mL)*, (*n* = 31)		62.8 (23.0–141.0)
Positive autologous serum test, *n* (%), (*n* = 30)		16 (53)

* Median (Interquartile Range, 25%–75%).

### 3.2 Changes in the urticaria activity and CU-Q2oL scores after low salicylate diet

**Figure 2:**
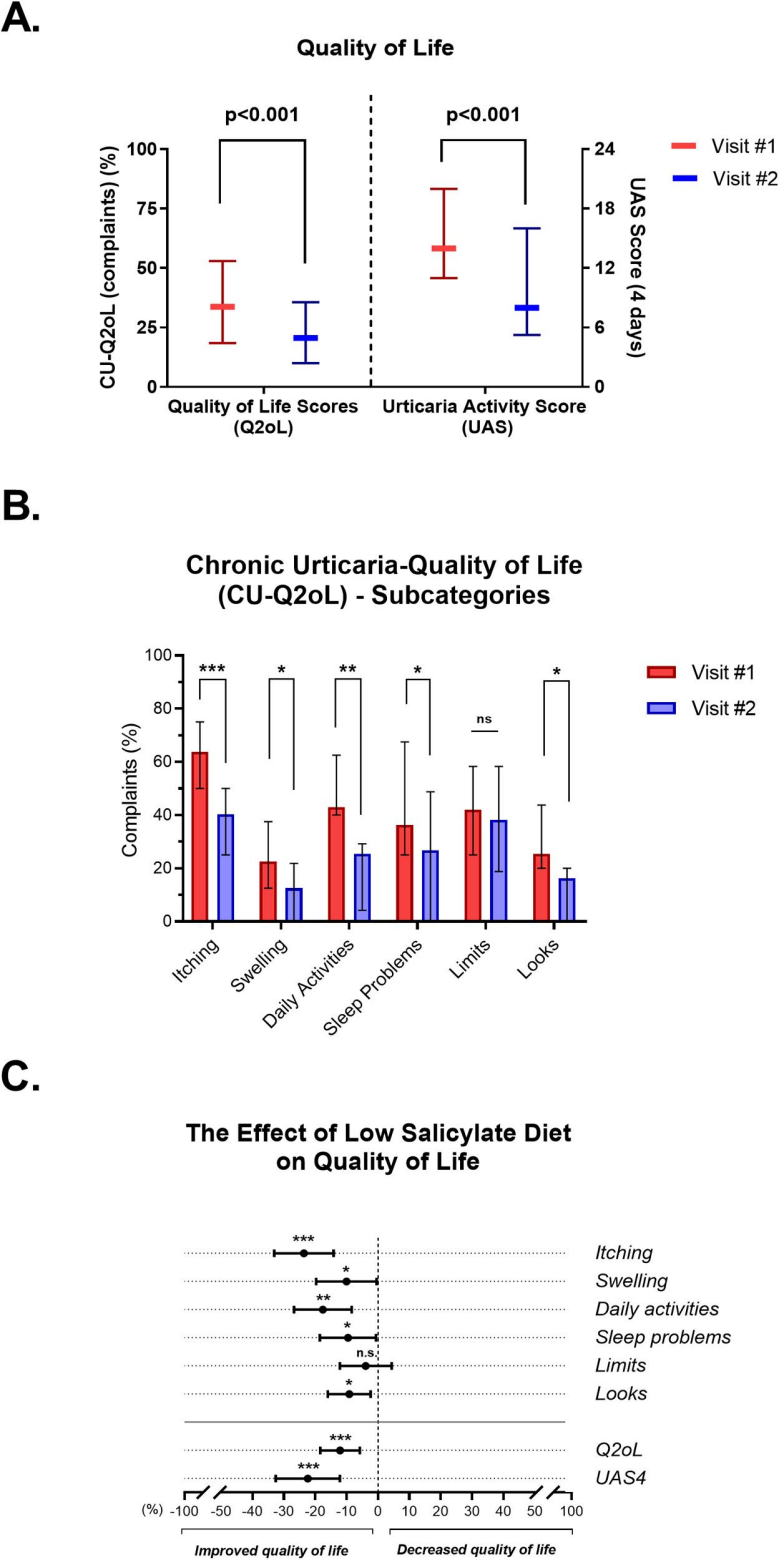
The effect of the low salicylate diet on symptom complaints. **(A)** CU-Q2oL complaints shown as percentages (lower score means less complaint). Urticaria Activity Scores recorded for 4 days. **(B)** Subcategories of CU-Q2oL, **(C)** Change on the quality of life after low salicylate diet (calculated as visit 2 scores minus visit 1 scores). (*n* = 36). n.s., not significant; *: *p* = 0.05 > *p* > 0.01, **: *p* = 0.01 > *p* > 0.001, ***: *p* = 0.001 > *p* > 0.0001.

The CU-Q2oL score decreased from 33.7% to 20.6% and UAS4 decreased from14 to 8 after the four-week low salicylate diet (*p* < 0.001 for each) (Figure 2A).

The subcategories of CU-Q2oL with significant changes after the low salicylate diet included itching (*p* < 0.001), swelling (*p* = 0.011), daily activities (*p* = 0.001), sleep problems (*p* = 0.038) and looks (*p* = 0.014) (Figure 2B). Only the subcategory named “limits” representing the limitation in daily activities did not change after low salicylate diet (*p* = 0.492). The quality of life significantly increased after the salicylate elimination diet (*p* < 0.001) (Figure 2C).

### 3.3 Plasma salicylic acid level

**Figure 3:**
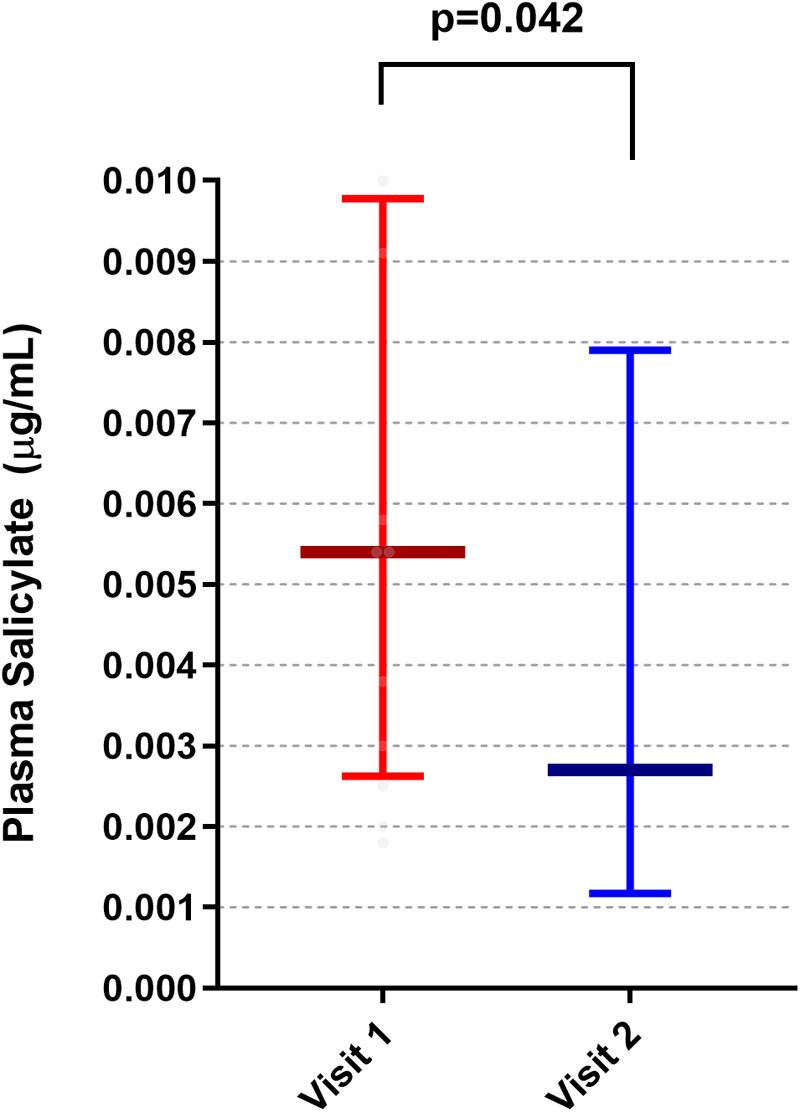
The effect of the low salicylate diet on plasma salicylate levels.

The blood salicylate level significantly decreased from 0.0054 µg/mL to 0.0027 µg/mL after the low salicylate diet (*p* = 0.042) (Figure 3). The correlation analysis between the salicylate levels and UAS4, CU-Q2oL, and subcategories including itching, swelling, daily activities, sleep problems, and looks-were not significant at baseline and after four weeks of low salicylate diet.

The comparison of the level of salicylate was similar between children and adults before and after low salicylate diet (Supplementary Table S2).

In the group of “no responder to treatment of antihistamine and/or Omalizumab” the level of salicylate did not change after low salicylate diet (before low salicylate diet: 0.0195 vs. after low salicylate diet: 0.0122; *p* = 0.655) but in the group of “partial responder to treatment of antihistamine and/or Omalizumab” the level of salicylate reduced significantly after low salicylate diet (before low salicylate diet: 0.0054 vs. after low salicylate diet: 0.0026; *p* = 0.015).

### 3.4 Urine PGE2, TXA2, PGF2α, LTE4 levels

**Figure 4:**
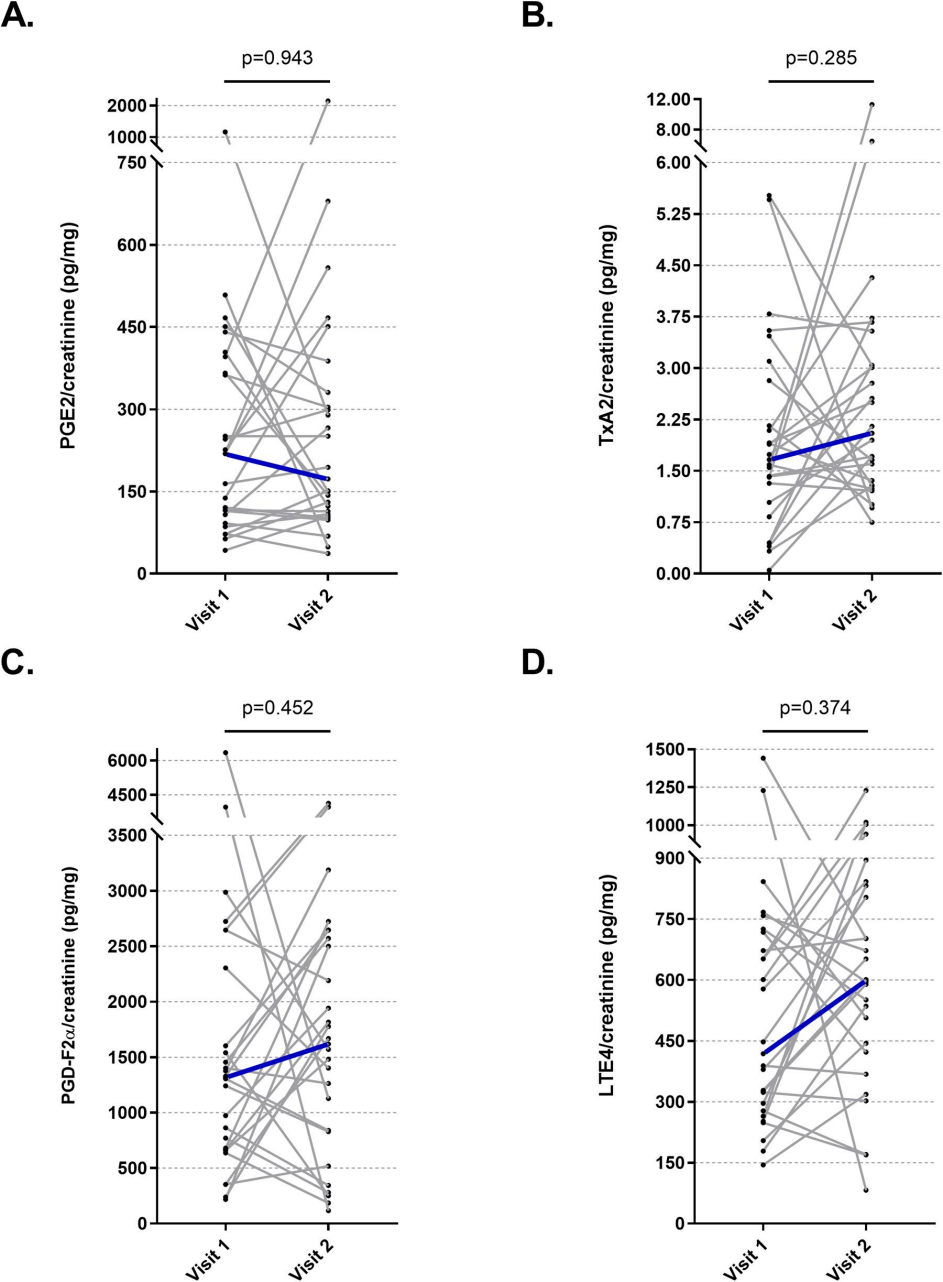
The effect of the low salicylate diet on urinary mediator levels. **(A)** Prostaglandin-E2, **(B)** Prostaglandin-F2α, **(C)** Thromboxane-A2, **(D)** Leukotriene-E4.

The urine PGE2, TXA2, PGF2α, and LTE4 levels did not change after the low salicylate diet (Figure 4). The urine PGE2, TXA2, PGF2α, and LTE4 levels did not show significant change in subgroups of children and adults after low salicylate diet (data not shown).

## 4 Discussion

We observed that patients on a low salicylate diet in our study reported that their CSU symptoms improved. They also reported that health quality in all parameters, including itching, swelling, daily activities, limits, looks, and sleep problems improved after the diet. Participants' compliances with the low salicylate diet were confirmed by the low blood salicylic acid level after the diet intervention. Patients also reported that the low salicylate diet was easy-to follow as an add-on therapy in CSU. Urinary metabolites, including PGE2, TXA2, PGF2α, and LTE4 levels of each participant, did not show a significant change between the baseline (visit 1) and after 4 weeks of a low salicylate diet (visit 2).

CSU is frequently treatment-resistant and long-standing, which is very challenging for patients. Literature suggests that specific dietary changes may be helpful in a subset of patients (16). While diet is not a first-line or universal recommendation for all chronic urticaria patients in the EAACI guidelines, it is recognized as a potential adjunctive strategy for those who may have specific food intolerances or pseudoallergy (1). Diet is a safe and low-cost treatment option that may help patients to decrease the attack frequency in CSU. However, there are different kinds of diets such as pseudoallergen-free diet, low-histamine diet, or diet without fish products (6, 16). The duration of diets is variable between 2 to 6 weeks. Therefore, which subgroups benefit from dietary interventions, which types of diets are effective, and the appropriate duration remain controversial, and the level of evidence is low for the benefit of diet modifications in CSU (16).

In the study by Kęszycka PK et al. 20 of 23 CSU patients (86.9%) reported a remission of symptoms of urticaria after a low salicylate diet (6). In 2 patients (8.7%), the severity of symptoms did not change, while 1 patient reported an increase in pruritus symptoms (6). Of the 23 CSU patients, 2 of them had only urticaria, 1 had CSU and asthma, and the remaining 13 patients had CSU, asthma and rhinosinusitis. All patients had aspirin hypersensitivity. The restrictive diet was followed only for 2–4 weeks. In this study, the food-item itch questionnaire (FIIQ) was used to assess remission of urticaria. The test was performed 3 times before the dietary intervention, and 3 times during the last 3 days of the intervention. The mean of 3 repetitions was used to compare the results before and after the diet. To the best of our knowledge, our study is the first study that investigated the effect of low dietary salicylate on blood salicylate level and urticaria severity. We used activity and health quality scores to evaluate the severity of CSU. We included all CSU patients independently of aspirin sensitivity evaluation. Our results were consistent with those of the study by Kęszycka PK et al. (6) in which low salicylate diet significantly improved health quality and symptom control in CSU patients with or without aspirin sensitivity after 4 weeks of low salicylate diet.

Lawrence et al. compared the amounts of salicylic acid excreted daily in the urine of non-vegetarians and vegetarians not taking salicylate drugs, and patients taking 75 or 150 mg aspirin/day, in their study (17). They showed that salicylic acid was significantly excreted more by vegetarians compared to non-vegetarians (17). The median amounts of salicylic acid excreted by vegetarians and the patient's taking aspirin were not significantly different (17). In Blacklock et al. study, median concentrations of salicylate levels in serum samples from vegetarians and non-vegetarians were found as 0.11 µmol/L and 0.07 µmol/L, respectively while the median serum concentration of salicylic acid in patients taking 75 mg daily aspirin was 10.03 µmol/L (8). In our study, the blood salicylate level was significantly decreased after the low salicylate diet. Purely vegetable diets provide less than 6 mg/day of salicylate and Janssen et al. concluded that this amount is probably too low to influence disease risk (18). However, in cell culture studies, it has been shown that salicylic acid can inhibit the cyclooxygenase pathway even at very low concentrations such as 0.1 µmol/L (9).

Cysteinyl leukotrienes play an important role in the pathogenesis of chronic urticaria and studies show that cysteinyl leukotrienes are one of the main mediators of aspirin induced urticaria (1). In Di Lorenzo et al. study, urine samples from patients with aspirin-induced urticaria had a significantly higher level of leukotriene release after the aspirin challenge test compared to patients with chronic urticaria without aspirin allergy and healthy controls (19). In Akoglu et al. study, a 4-week pseudoallergen diet reduced the urine LTE4 levels. This reduction was found significantly correlated with the change in mean UAS (20). In the present study, no change was observed in urine metabolite levels after a 4-week low salicylate diet. Plasma salicylate level was very low after the salicylate restriction diet and this reduced salicylate levels may have an insufficient effect on urine metabolite levels. Urticaria is a predominantly mast cell–driven disease and T cells, eosinophils, basophils, and other cells which play a role in complex nature of the pathogenesis of urticaria (1). Diet salicylate may affect urticaria pathogenesis via different pathways such as basophil activation out of the cysteinyl leukotriene pathway. This may have a negative impact on urine metabolite levels due to different mechanisms. According to the updated classification of allergic diseases and hypersensitivity reactions, type VII represents direct cellular and inflammatory response to chemical substances (21). Idiosyncratic reactions include cross-reactive hypersensitivity to NSAID, and NSAIDS-exacerbated cutaneous disease (NECD), in patients with underlying CSU is classified in the new type VII hypersensitivity reaction. Since a significant association with the release of eicosanoid mediators was not observed in this study, it is possible to speculate that the underlying mechanism in the natural food sourced salicylate induced urticaria exacerbation may not be linked to the inhibition cyclooxygenase-1 (COX-1) and the production of LTs, PGs and TXs. Furthermore, while the administration of a low salicylate diet resulted in a significant reduction in both blood salicylate levels and UAS4 over a four-week period, a notable correlation between the UAS4, CU-Q2oL, and blood salicylate levels was not established. This finding suggests that the therapeutic effects of the low salicylate diet on the severity of CSU may be attributed to factors other than salicylate itself, potentially implicating different compounds present in the dietary constituents. However, the potential contribution of the placebo effect and a reduction in systemic inflammation due to lowered salicylate levels should also be considered.

The first limitation of the study is, even though the study had planned to perform cross-over with a period of low intake of salicylate diet and then with a free diet in a high number of participants, the restrictions and shutdown of the COVID-19 pandemic prevented the inclusion of a high number of patients, and the third and fourth visit of most of our participants in which it was planned they would have a diet with high salicylate content. Another limitation of the study is the lack of *in vitro* experiments such as basophil activation test and lymphocyte activation/proliferation tests by salicylate which would help to reveal the underlying mechanism of salicylate sourced from foods inducing mast cells and basophils. The other limitation is whether the patients are hypersensitive to NSAIDs by performing aspirin challenge tests.

In conclusion, a low salicylate diet may help to decrease the severity of urticaria and improve the quality of life. However, further research with larger patient numbers is required to clarify whether the effect observed in our study is reproducible.

## Data Availability

The raw data supporting the conclusions of this article will be made available by the authors, without undue reservation.
