# Supplementary material for: Effect of low salicylate diet and blood salicylate level on the symptom control of chronic spontaneous urticaria
Source: Front Allergy. 2025 Dec 8;6:1687600. doi: 10.3389/falgy.2025.1687600 (PMC12719075; doi:10.3389/falgy.2025.1687600)
Supplement: Supplementary file 1 [file Table1.docx]

**Supplementary Table 1.** Urticaria activity score**^1^**

**
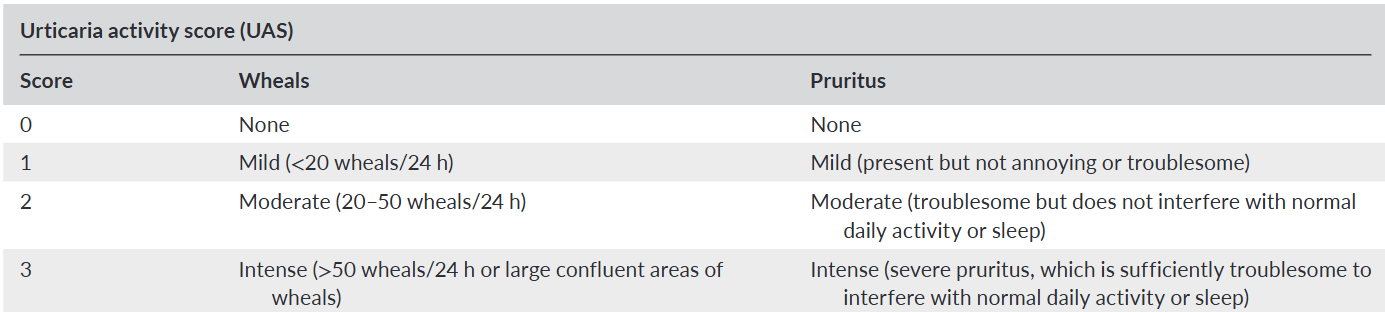
**

**References:**

1. Zuberbier, T., Abdul Latiff, A. H., Abuzakouk, M., Aquilina, S., Asero, R., Baker, D., et al. (2022). "The international EAACI/GA(2)LEN/EuroGuiDerm/APAAACI guideline for the definition, classification, diagnosis, and management of urticaria". Allergy, 77(3), 734-766. <https://doi.org/10.1111/all.15090>.
