# Supplementary material for: Effect of low salicylate diet and blood salicylate level on the symptom control of chronic spontaneous urticaria
Source: Front Allergy. 2025 Dec 8;6:1687600. doi: 10.3389/falgy.2025.1687600 (PMC12719075; doi:10.3389/falgy.2025.1687600)
Supplement: Supplementary file 2 [file Table2.docx]

**Supplementary Table 2.** Changes in salicylate levels in children and adults separately.

|  | **Children** | | | **Adults** | | |
| --- | --- | --- | --- | --- | --- | --- |
|  | **Visit-1** | **Visit-2** | **P*** | **Visit-1** | **Visit-2** | **P*** |
| **Level of salicylate** (Median-Interquartile range) | 0.058  (0.0025-0.04236) | 0.0028  (0.0026-0.0084) | 0.086 | 0.0039  (0.0025-) | 0.0007  (0.0007- ) | 0.180 |

*Paired t-test (Wilcoxon test) is used to compare the groups

|  | **Visit-1** | | | **Visit-2** | | | |
| --- | --- | --- | --- | --- | --- | --- | --- |
|  | **Children** | **Adults** | **P*** | | **Children** | **Adults** | **P*** |
| **Level of salicylate** (Median-Interquartile range) | 0.058  (0.0025-0.04236) | 0.0039  (0.0025-) | 0.480 | | 0.0028  (0.0026-0.0084) | 0.0007  (0.0007- ) | 0.097 |

*T-test is used to compare the groups
